# **Horizontal hexagonal six-stitch pancreaticojejunostomy (HEXA-PJ): a simple and reproducible anastomotic technique**

**DOI:** 10.1007/s00595-026-03322-z

**Published:** 2026-05-22

**Authors:** Asahi Sato, Kazuyuki Nagai, Kei Yamane, Satoshi Nomura, Kentaro Kadono, Takahiro Nishio, Katsunori Sakamoto, Satoshi Ogiso, Yoichiro Uchida, Takashi Ito, Takamichi Ishii, Etsuro Hatano

**Affiliations:** Department of Surgery, Graduate School of Medicine, Kyoto University, 54 Shogoinkawaharacho, Sakyo-ku, Kyoto, 606-8507 Japan

**Keywords:** Pancreaticoduodenectomy, Duct-to-mucosa, Pancreatic fistula

## Abstract

Pancreaticoduodenectomy (PD) is one of the most technically demanding gastrointestinal surgical procedures. Among its various components, pancreaticojejunostomy (PJ) is particularly complex. We developed a simple and easy-to-perform pancreaticojejunostomy technique, termed Horizontal Hexagonal Six-stitch Pancreaticojejunostomy (HEXA-PJ). After placing U-shaped sutures on the jejunal seromuscular layers and pancreas, the HEXA-PJ technique begins with the placement of two anterior traction sutures on the pancreatic duct, followed by suturing at the 3- and 9-o’clock positions. Owing to the improved visualization achieved through this sequence, the remaining posterior sutures can be easily placed. After completing the anterior sutures, the duct-to-mucosa anastomosis is finalized in a controlled manner. Finally, modified Blumgart sutures were placed and ligated. This structured approach can be easily learned and can shorten the anastomotic time of the PJ without increasing the risk of significant postoperative complications.

**Supplementary Information:**

The online version contains supplementary material available at 10.1007/s00595-026-03322-z.

## Introduction

 Pancreatoduodenectomy (PD) is the only curative surgical treatment for tumors arising in the periampullary region. Despite substantial improvements in perioperative management and surgical outcomes, PD remains one of the most invasive abdominal surgeries [1–3]. PD is a highly complex procedure involving multiple operative steps and often a prolonged operative time.

Among the reconstructive steps, pancreatoenteric anastomosis is one of the most critical and technically demanding. Numerous techniques have been developed to reduce the incidence of postoperative pancreatic fistula (POPF). However, no single method has demonstrated either any clear superiority or gained universal acceptance [4–8]. Furthermore, accumulating evidence suggests that POPF is influenced more strongly by patient-related factors than by surgical techniques alone [9, 10]. Therefore, beyond safety alone, an ideal pancreaticojejunostomy (PJ) technique should also be simple, reproducible, and easy to standardize for broader surgical practice and training purposes. However, many reported techniques rely heavily on individual expertise and technical nuances, which may limit their generalizability to other laboratories.

Against this background, we developed a novel pancreaticojejunostomy technique, termed Horizontal Hexagonal Six-Stitch Pancreaticojejunostomy (HEXA-PJ), designed to prioritize simplicity and reproducibility while maintaining procedural safety.

## Methods

### Patients

Patients who underwent PD at Kyoto University Hospital between October 2023 and September 2025 were retrospectively reviewed. The clinical outcomes of the HEXA-PJ technique were compared with those of conventional PJ. Minimally invasive (laparoscopic and robotic) PD cases were excluded. This study was conducted in accordance with the Declaration of Helsinki and approved by the Institutional Review Board of Kyoto University (R1721-3).

PD is performed using any of the classic methods, depending on the extent of the disease [11]. After dissection of the mesenteric lymph nodes, Kocher’s mobilization, and dissection of the hepatoduodenal ligament, the pancreas was divided. The pancreatic parenchyma was divided using electrocautery, whereas the tissue surrounding the pancreatic duct was sharply divided with a scalpel. The nerve plexus around the pancreatic head was then dissected. If the portal or superior mesenteric vein showed malignant invasion, combined resection and reconstruction of the vessels were performed using 6 − 0 polypropylene sutures. After completing PJ, as described below, choledochojejunostomy and antecolic reconstruction of the alimentary tract were performed. Two or three closed suction tubes were placed behind the biliary anastomosis and around the pancreatic anastomoses.

The details of representative perioperative management (such as the administration of antibiotics, blood sampling, fluid drainage, and drain removal) have been previously described [11].

## Horizontal Hexagonal six-stitch pancreaticojejunostomy (HEXA-PJ)

**Fig. 1:**
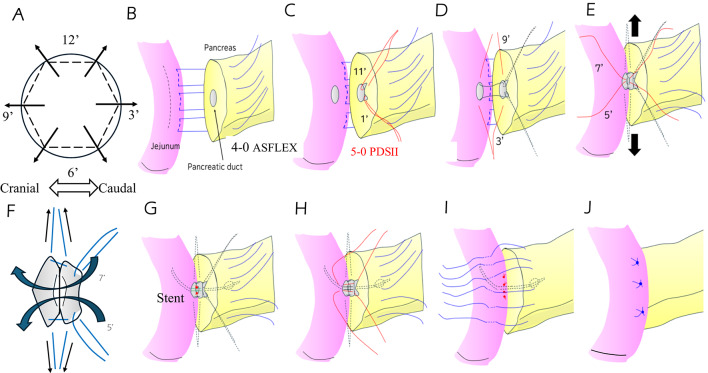
Diagrams of Horizontal Hexagonal Six-stitch Pancreaticojejunostomy (HEXA-PJ). A clock-face analogy is used for the description of duct-to-mucosa anastomosis (**A**). The U-shaped sutures using 4 − 0 polyvinylidene difluoride threads in accordance with the modified Blumgart method are placed **(B**). Double-armed 5 − 0 polydioxanone sutures are placed at the 11- and 1-o’clock positions of the pancreatic duct (**C**). The 3- and 9-o’clock sutures (**D**). With appropriate traction at the 3- and 9-o’clock positions (arrows), the posterior walls are easily aligned, and 5- and 7-o’clock are smoothly handled (**E**,** F**). The 5- and 7-o’clock sutures are tied first, and the pancreatic duct stent is positioned and secured using one of these sutures (**G)**. The 11- and 1-o’clock sutures ae completed, and all duct-to-mucosa sutures are tied (**H**). The anterior seromuscular layer of the modified Blumgart sutures is placed and ligated to complete (**I**,** J**)

**Fig. 2:**
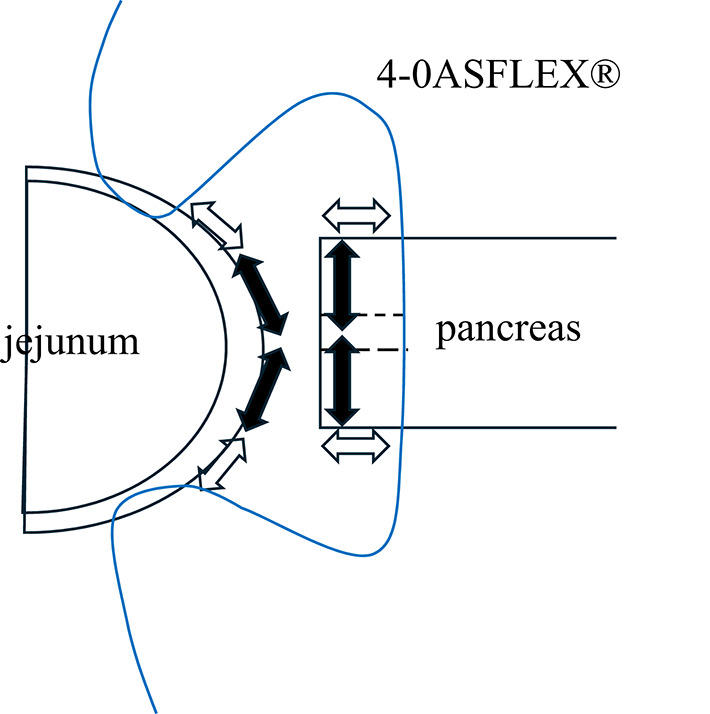
A cross-sectional diagram of the modified Blumgart anastomosis

HEXA-PJ is a pancreatic duct-to-mucosa anastomosis combined with the modified Blumgart technique (Supplemental Video) [5]. In the following description, the orientation of the duct-to-mucosa anastomosis is described using a clock-face analogy (Fig. 1A). After removal of the specimen, the jejunal limb was brought up in a retrocolic manner. For the posterior seromuscular layer of the jejunum, three double-armed 4 − 0 polyvinylidene difluoride sutures (4-0ASFLEX, ASSP504-OⅡN, CROWNJUN, Chiba, Japan) were placed longitudinally and then passed through the pancreatic parenchyma (Fig. 1B). Subsequently, double-armed 5 − 0 polydioxanone sutures (5-0PDSⅡ, Z867H, ETHICON, NJ, USA) were placed at the 11- and 1-o’clock positions of the pancreatic duct as traction sutures (Fig. 1A, C). Additional sutures were then placed at the 3- and 9-o’clock positions, followed by the 5- and 7-o’clock positions (Fig. 1D, E). With appropriate traction at the 3- and 9-o’clock positions, the posterior wall of the pancreatic duct and the jejunal enterotomy were easily aligned, allowing smooth needle handling (Fig. 1F). The 5- and 7-o’clock sutures were tied first, and the pancreatic duct stent was positioned and secured using one of these sutures (Fig. 1G**)**. The inner arms of the 11- and 1-o’clock sutures were then passed through the jejunal opening and tied carefully to avoid suture rupture (Fig. 1H). Finally, the anterior layer of the modified Blumgart sutures was placed and ligated to complete the anastomosis (Fig. 1I, J). The jejunal seromuscular sutures should be placed on the antimesenteric side at a distance corresponding to half of the pancreatic thickness plus the length of the transpancreatic sutures, thus allowing the jejunum to naturally cover the pancreatic stump (Fig. 2).

Conventional duct-to-mucosa anastomosis was performed with eight interrupted sutures using 6 − 0 polypropylene (6 − 0 PROLENE, EH7400H, ETHICON, NJ, USA), with all knots positioned outside the lumen. After partial ligation and stent insertion secured by an inner absorbable suture, the remaining sutures were placed and tied. The modified Blumgart anastomosis was completed as described above.

As a principle, an internal pancreatic duct stent using a lost stent was placed in all patients; however, in cases with a dilated main pancreatic duct or firm pancreas, omission of stent placement was permitted at the surgeon’s discretion.

## Definition of complications

Postoperative pancreatic fistula (POPF), post-pancreatectomy hemorrhage (PPH), delayed gastric emptying (DGE), and post-pancreatectomy acute pancreatitis (PPAP) were diagnosed according to the criteria of the International Study Group for Pancreatic Surgery [12–15]. Other complications were recorded when the Clavien-Dindo classification grade was greater than III [16].

### Statistical analysis

The JMP software program version 13.0 (JMP Statistical Discovery, Cary, NC, USA) was used for all statistical analyses. Continuous variables are presented as the mean ± standard deviation (SD) values unless otherwise indicated and were compared using the Mann-Whitney U-test. Categorical variables were compared using the chi-squared or Fisher’s exact tests. Statistical significance was set at *p* < 0.05.

## Results

During the study period, 112 PD were performed. Minimally invasive procedures (laparoscopic, *n* = 29; robotic, *n* = 13) and simultaneous liver resection (*n* = 2) were excluded from the analysis. Therefore, 68 consecutive patients who underwent open PD were analyzed. There were 21 cases of the HEXA-PJ technique and 47 cases of the conventional method. The choice of duct-to-mucosa reconstruction was left to the discretion of the surgeon. All surgeons had more than 15 years of experience and were board-certified as gastrointestinal surgeons.

The demographic characteristics and perioperative parameters are presented in Table 1. The baseline characteristics, such as age, sex, body mass index, and performance status, were comparable between the two groups. Pancreatic ductal adenocarcinoma was observed in 10 (48%) and 32 (68%) patients in the HEXA-PJ and conventional groups, respectively.

**Table 1:** Comparison of the demographic and operation-related parameters between HEXA-PJ and the conventional method

	Hexa-PJ (*N*=21)	Conventional (*N*=47)	*P*
Demographic factors			
Age [y/o], median (range)	75 (38-86)	72 (37-87)	0.915
Sex, female (%)	5 (24)	23 (49)	0.052
ASA-PS 1/2/3	0/16/5	1/40/6	0.431
Body mass index	21.3 ± 3.0	22.5 ± 4.3	0.297
Diseases			0.109
PDAC	10 (48)	32 (68)	
Others	11 (52)	15 (32)	
Neoadjuvant therapy (%)	8 (36)	26 (55)	0.189
Operation-related factors			
Soft pancreas (%)	8 (38)	22 (47)	0.504
Operative time [min]	458.9 ± 99.4	426.3 ± 76.4	0.167
EBL [ml]	679.4 ± 482.6	693.9 ± 635.3	0.676
Transfusion (%)	2 (10)	6 (13)	0.701
Stents (%)	19 (91)	44 (94)	0.647
MPD diameter	5.4 ± 2.7	4.4 ± 2.0	0.192
1POD serum amylase	477.9 ± 561.5	501.4 ± 495.3	0.691
1POD drain amylase	2749.1 ± 3839.2	5597.6 ± 14281.7	0.974
3POD serum amylase	109.8 ± 131.2	92.2 ± 866.8	0.533
3POD drain amylase	923.0 ± 2166.7	999.7 ± 2899.2	0.582
Time of PJ anastomoses [min]	25.6 ± 7.2	33.2 ± 10.4	0.004*
CR-POPF (%)	3 (14)	5 (11)	0.666
Grade BL/ B/ C	3/3/0	10/5/0	0.751
PPH, grade B/ C	0/0	4/0	0.168
DGE, grade B/ C	3/0	4/0	0.469
PPAP (%)	4 (19)	11 (23)	0.689
CD≥3 (%)	4 (19)	8 (17)	0.840
Grade 3a/3b/4/5	4/0/0/0	6/2/1/0	0.630
IVR (%)	2 (10)	4 (9)	0.892
Re-operation (%)	0 (0)	2 (4)	0.337
Duration of intravenous antibiotics [days]	7.5 ± 5.6	9.0 ± 6.6	0.433
Use of TAZ/PIPC (%)	6 (29)	26 (55)	0.041*
LOS [days]	21.1 ± 8.4	22.7 ± 10.0	0.447
Readmission (%)	1 (5)	3 (7)	0.800

PDAC; Pancreatic ductal adenocarcinoma, BTC; Biliary tract cancers, EBL; Estimated blood loss, MPD; Main pancreatic duct, POD; Postoperative day, PJ; Pancreato-jejunal, CR-POPF; Clinically relevant postoperative pancreatic fistula, BL; Biochemical leakage, PPH; Post-pancreatectomy hemorrhage, DGE; Delayed gastric emptying, PPAP; Post-pancreatectomy acute pancreatitis, CD; Clavien-Dindo classification, IVR; Interventional radiology, LOS; Length of hospital stay, TAZ/PIPC; tazobactum/piperacillin

**Table 2:** Comparison of HEXA-PJ and the conventional method stratified by the main pancreatic duct diameter (**A**) and the pancreatic texture (**B**). MPD; Main pancreatic duct, CR-POPF; clinically relevant postoperative pancreatic fistula, PJA; Pancreatico-jejunal anastomosis

A									
MPD diameter	MPD ≤ 3 mm		p	3 mm < MPD ≤ 6 mm		p	MPD≥ 6 mm		p
	HEXA-PJ (n=5)	Conventional (n=16)		HEXA-PJ (*n*=10)	Conventional (n=24)		HEXA-PJ (n=6)	Conventional (n=7)	
CR-POPF [%]	2 (40)	4 (25)	0.517	1 (10)	0 (0)	0.116	0 (0)	1 (14)	0.335
Time of PJA [min]	30.4 ± 6.9	35.9 ± 8.1	0.172	22.8± 7.1	32.7 ± 12.6	0.010*	25.7 ±6.5	29.0 ± 4.7	0.387

B						
Pancreatic texture	Soft		p	Hard or intermediate		p
	HEXA-PJ (n=8)	Conventional (n=22)		HEXA-PJ (n=13)	Conventional (n=25)	
CR-POPF [%]	3 (38)	5 (23)	0.418	0 (0)	0 (0)	1.000
Time of PJA [min]	26.8 ± 9.7	34.0 ± 7.4	0.067	24.8± 5.4	32.6 ± 12.5	0.029*

The anastomotic time was significantly shorter in the HEXA-PJ group than in the conventional group (25.6 ± 7.2 vs. 33.2 ± 10.4 min, *p* = 0.004). The prevalence of a soft pancreas and the diameter of the main pancreatic duct were not significantly different. No significant differences were observed in the incidence of POPF (14% vs. 11%, *p* = 0.666), PPH (0% vs. 9%, *p* = 0.168), DGE (14% vs. 9%, *p* = 0.469), PPAP (19% vs. 23%, *p* = 0.689), or Clavien–Dindo grade III or higher complications (19% vs. 17%, *p* = 0.840) (Table 2).

To further clarify the utility of the HEXA-PJ technique, we performed a stratified analysis according to the pancreatic duct diameter and texture. In both the HEXA-PJ and conventional groups, the PJ anastomosis time was the longest in patients with a duct diameter of ≤ 3 mm. Across all duct diameter categories, the PJ anastomosis time tended to be shorter with HEXA-PJ than *with the conventional technique.* Regarding pancreatic texture, the PJ anastomosis time was significantly shorter with HEXA-PJ in patients with hard or intermediate pancreas, whereas no significant difference was observed in those with soft pancreas. These results suggest that a small pancreatic duct and soft pancreatic texture may represent the relative technical limitations of the HEXA-PJ technique.

Finally, an exploratory analysis was performed to determine whether board certification in hepatobiliary-pancreatic surgery influenced the PJ anastomotic time for each technique. In the HEXA-PJ group, the mean anastomosis time was 21.1 ± 2.4 min for HPB board-certified surgeons (*n* = 5) and 27.0 ± 7.8 min for non-board-certified surgeons (*n* = 16; *p* = 0.054). In the conventional group, the anastomotic time was 29.8 ± 10.5 min when performed by board-certified surgeons (*n* = 5) and 33.7 ± 10.4 min when performed by non-board-certified surgeons (*n* = 42). In addition, among non-board-certified surgeons, the anastomotic time tended to be shorter with HEXA-PJ than with the conventional technique, although the difference was not statistically significant (*p* = 0.055).

## Discussion

HEXA-PJ was designed to simplify the duct-to-mucosa PJ that we routinely perform and to improve its reproducibility. The important aspects of this technique are as follows: (1) a fixed six-stitch hexagonal duct-to-mucosa configuration, (2) avoidance of the 12 and 6 o’clock positions, (3) initial traction sutures at 11 and 1 o’clock, (4) cranio-caudal traction to facilitate suturing of the posterior wall, and (5) a predefined sequence of stitch placement to improve posterior wall exposure and procedural flow.

The present HEXA-PJ is a unique technique in which sutures are placed in a hexagonal configuration without suturing at the 12- and 6-o’clock positions, thereby improving the handling of sutures by assistants and visualization of the posterior wall. In addition, the HEXA-PJ method uses fewer sutures than the conventional technique, which is also considered to contribute to procedural simplicity and reduced risk of pancreatic parenchymal injury. Although these factors are difficult to quantify objectively, they may contribute to smoother suture handling and a more consistent procedural flow. Such qualitative advantages may be particularly important in complex procedures, such as PD, in which a stable and reproducible anastomotic workflow is desirable. Importantly, these features were associated with a reduction in anastomotic time during HEXA-PJ without increasing the occurrence of significant postoperative complications.

The validity of reducing the number of duct-to-mucosa sutures from eight to six may be questioned. While fewer sutures may facilitate suture handling, whether six stitches provide adequate approximation in cases with a markedly dilated duct, such as intraductal papillary mucinous neoplasm, remains unclear. However, in our subgroup analysis of patients with a dilated pancreatic duct, the incidence of postoperative pancreatic fistula did not increase in the HEXA-PJ group, despite the reduced number of sutures. In our limited experience, no additional sutures were required for the repair. However, this observation alone does not establish the universal adequacy of the six-stitch configuration. One potential advantage of HEXA-PJ is that its good visibility and balanced suture placement make intraoperative adjustment straightforward, allowing the addition of reinforcing sutures at the surgeon’s discretion when necessary. Further accumulation of cases is needed to validate this approach, particularly in patients with a dilated pancreatic duct.

Although duct-to-mucosa anastomosis was once shown to be superior in a randomized controlled trial [17], many subsequent studies have continued to compare this technique with invagination methods, thus reflecting the persistent lack of consensus regarding the optimal PJ technique [18–21]. The continued interest in invagination techniques may stem from concerns about the technical complexity and limited reproducibility of duct-to-mucosa anastomosis, particularly in routine clinical practice. Using a porcine model, Yang et al. analyzed the healing process of cholangiojejunostomy and demonstrated that the epithelial layers were closely connected earlier, whereas abundant cicatricial connective tissue existed beneath the mucosa [22]. Based on these findings, Wu et al. proposed the hypothesis of “mucosal priority healing” in duct-to-mucosa anastomosis and demonstrated that the rate of severe complications was lower in the duct-to-mucosa group [23]. Taken together, these findings support the theoretical importance of duct-to-mucosa anastomosis, despite its technical complexity.

HEXA-PJ is combined with the established modified Blumgart anastomosis (mBA), introduced by Fujii et al. [5]. The theoretical advantages of mBA include the obliteration of dead space at the pancreatic stump and longitudinal fixation of the jejunum, which may reduce shear stress on the pancreatic parenchyma and avoid excessive tension that could lead to parenchymal tearing. Although numerous studies have reported the use of mBA, we believe that the technique has not been fully standardized across institutions or surgeons [4, 24]. In the process of establishing HEXA-PJ, we also sought to enhance the reproducibility of mBA by theoretically defining the needle placement on the jejunal side, thereby enabling surgeons to perform anastomosis based on a consistent and shared conceptual framework. This structured design allows the procedure to be performed without hesitation and it may facilitate more efficient completion of anastomosis.

This study has several limitations. First, the sample size was relatively small because the primary aim was to report the preliminary results of this novel technique. Second, the retrospective design introduced inevitable heterogeneity in surgical procedures and perioperative management, which may have led to potential selection bias. For example, the observed reduction in tazobactam/piperacillin use may be attributable to multiple factors, including the refinement of prophylactic antibiotic strategies in collaboration with the infection control team and enhanced attention to antimicrobial stewardship. Third, because the two techniques differed not only in stitch number but also in suture material, the independent effect of suture material on outcomes could not be determined. In HEXA-PJ, 5 − 0 PDS was used because of its better visibility and lower tendency to become entangled. Fourth, although HEXA-PJ tended to reduce the anastomotic time even when performed by non-board-certified surgeons, the number of such cases was limited, and the difference was not statistically significant. Therefore, the effect of the surgeon experience on the utility of HEXA-PJ could not be fully evaluated in this study.

Future studies with larger cohorts and prospective validation are needed to clarify the true utility of HEXA-PJ, particularly because this technique is inherently simple, time-efficient, cost-effective, and potentially well-suited for adoption by less-experienced surgeons. Nevertheless, given its simplicity and reproducibility, the HEXA-PJ technique may be suitable for broader adoption and future multicenter studies.

In conclusion, the HEXA-PJ technique is a feasible and efficient approach for PJ. Enhanced visualization and simplified needle handling by adopting a hexagonal suture configuration are key features that may contribute to improved reproducibility and the safety of anastomosis.

## Supplementary Information

Below is the link to the electronic supplementary material.

Supplementary Material 1
